# Wrestling game injuries among children in Dakar: a report on 172 cases

**DOI:** 10.11604/pamj.2017.26.150.7961

**Published:** 2017-03-15

**Authors:** Gabriel Ngom, Azhar Salim Mohamed, Papa Alassane Mbaye, Mbaye Fall, Oumar Ndour, Aimé Lakh Faye, Zakaria El-Hasnaoui

**Affiliations:** 1 Unit of Pediatric Surgery of the University Hospital Aristide Le Dantec in Dakar, Sénégal; 2 Service de Chirurgie Pédiatrique, Hôpital d’Enfants Albert Royer, Dakar, Sénégal; 3 Centre de Santé des HLM, Dakar, Sénégal

**Keywords:** Wrestling game, Injuries, Children, Fracture, Fall, Dakar

## Abstract

**Introduction:**

The objective was to report epidemiological and lesional features among children practicing wrestling as a game in Dakar, Senegal.

**Methods:**

It was a retrospective study including all patients under 16, victims of wrestling game injuries. We studied epidemiological and lesional aspects in children: frequency of wrestling game injuries among all games, age, sex, geographic origin, place of injury, parent’s socioeconomic status, nature of the injury and location.

**Results:**

Wrestling game injuries represented 19.9% injuries in all games. Sex-ratio was 33.4. The most affected age group was the 6-10 years old age group. The majority of children are from suburban Dakar (64%). Injuries occurred most often at home and in the street. Most children are from low socioeconomic status (64%). Fractures predominated and were localized almost exclusively on the elbow.

**Conclusion:**

Wrestling game injuries in Dakar occur among older children from the suburbs, living in the neighborhood of great wrestling champions’ districts of residence. Wrestling game cause injuries, consisting mostly of elbow fractures.

## Introduction

Four types of wrestling are practiced in the world, each with its specificity. These are: Olympic wrestling, freestyle wrestling, women's wrestling and traditional and folk wrestling [1, 2]. Senegalese wrestling is a traditional wrestling [2]. In Senegal, traditional wrestling was practiced in villages to celebrate the end of the harvesting season [3]. It aimed at assessing the strength of men before designating the village champion, the winner of the tournament [3]. Today, there is a special form of wrestling, which is only practiced in Senegal and finds its specificity in the acceptance of bear-hand boxing. This type of wrestling, which is operated for commercial purposes, has become a social phenomenon. Wrestlers can win up to 76,335 euros in a single fight. They represent their places of origin or residence, in the eyes of the population; and a social role model of success. Children practice this type of wrestling to impersonate their idols, to become like those wrestlers one day. They are exposed to trauma due to their fragility and their lack of technicity in this game. The aim of this study was to report the epidemiological and lesional aspects of accidents due to wrestling game, referred to the Pediatric Surgery Department of Aristide Le Dantec Hospital in Dakar, Senegal.

## Methods

This is a retrospective study conducted between January the 1st, 2009 and December the 31st, 2013. All patients under 16, victims of the wrestling game received at the Pediatric Surgery Department of Aristide Le Dantec Hospital in Dakar during that period were included in the study. Injuries which occurred during in training wrestling schools where children learn wrestling techniques were excluded. We evaluated the frequency of wrestling game injuries in the study period over all game, as compared to other game injuries which occurred during the study period. The following epidemiological parameters aspects were studied in each child: age, sex, geographic origin, place where the injury occurred and the parents’ socio-economic status. Children were divided into three age groups: 0-5, 6-10 and 11-15 years old. The socioeconomic status was credited as good, medium and low taking into account the standard of living of the parents rated by place of residence, income and expenditure per capita. We assessed geographic origin based on three areas: Central Dakar, area where there is the concentration of State services and businesses, Suburban Dakar and other areas from Senegal. Lesional aspects included the nature of the injury (contusion, dislocation, sprain, and fracture, wound) and the location of the lesion.

## Results

### Epidemiological aspects

**Figure 1:**
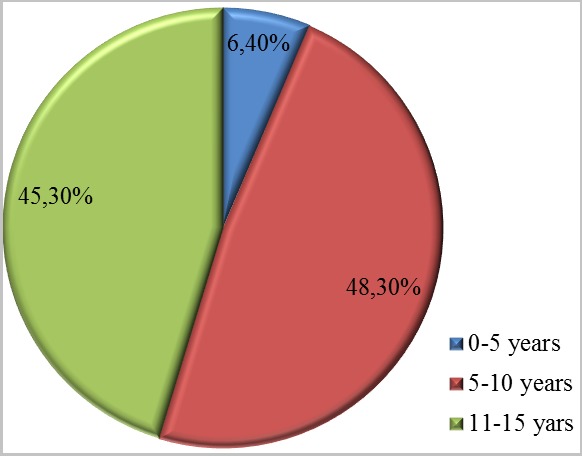
Distribution of wrestling game injuries to age groups

**Figure 2:**
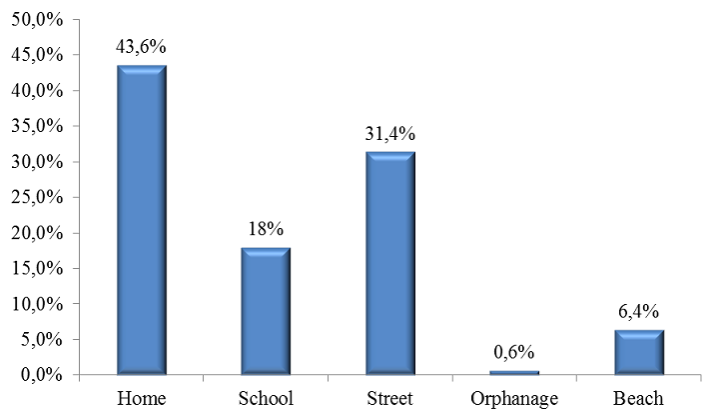
Distribution of wrestling game injuries by place of occurrence

**Figure 3:**
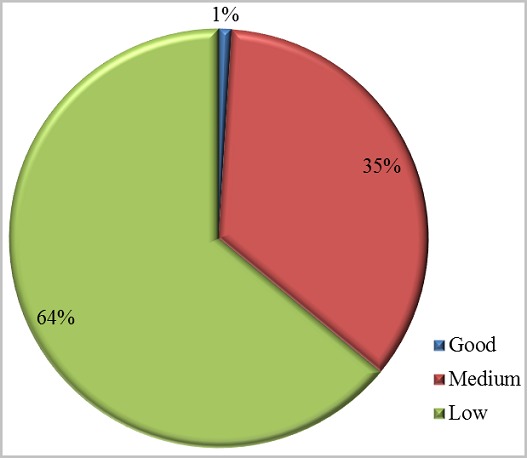
Distribution of wrestling game injuries by socio-economic level

One hundred and seventy-two cases of wrestling game injuries were identified among 863 cases of game injuries (19.9%). The series consisted of 167 boys and 5 girls (sex ratio of 33.4). The mean age was 9 and the most affected age group was the 6-10 years old group (Figure 1). The breakdown by geographical origin was: Central Dakar: 33%; Suburban Dakar: 64%, and other areas from Senegal: 3%. Accidents occurred most often at home and on the street (Figure 2). The children were from mostly of low socioeconomic group status (Figure 3).

### Lesional aspects

**Figure 4:**
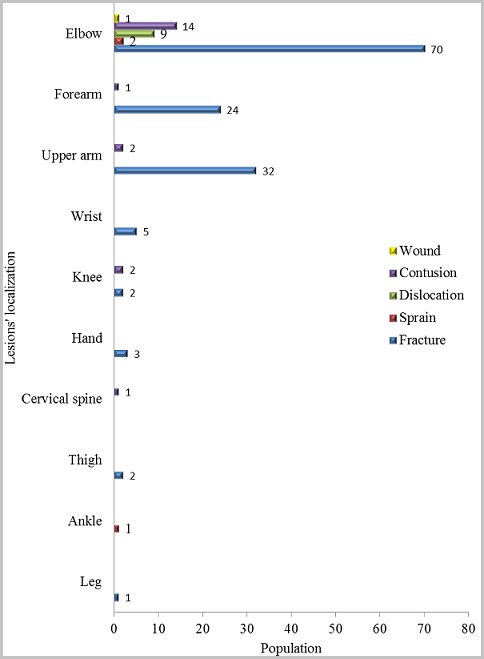
Distribution of lesions' type by localization

Lesions were localized almost exclusively on limbs (171 cases) and essentially on thoracic limbs (163 cases). Elbow lesions predominated, followed by forearm and shoulder lesions. Fractures largely come at the top of the lesions, followed by contusions and dislocations (Table 1). All fractures were closed fractures. There were 89 cases of diaphyseal fractures and 50 cases of epiphyseal fracture-separations. They prevailed at the humerus with 85 cases, consisting of 35 cases of supracondylar fractures, 30 cases of the medial epicondyle fractures and 20 cases of the humeral diaphysis fractures. Lesions’ description and localizations are shown in Figure 4. Contusions were predominant at the elbow with 72% of the cases. All dislocations were localized at the elbow (7 cases). Two sprains occurred at the elbow and one occurred at the ankle. A wound was located at the elbow.

**Table 1:** Lesions’ description

Lesion	Effective
Fracture	139
Contusion	20
Dislocation	9
Sprain	3
Wound	1

## Discussion

**Figure 5:**
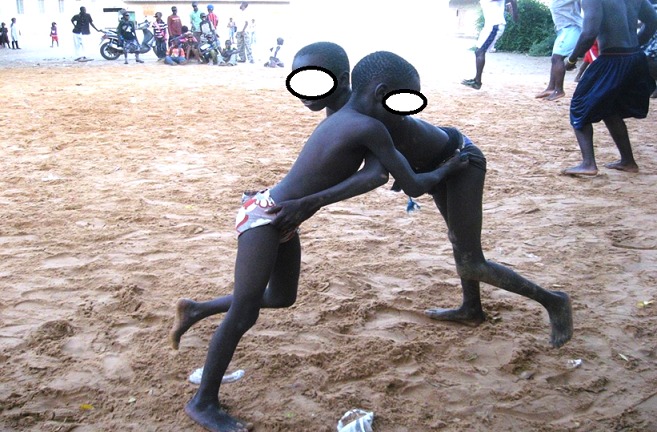
Children practicing wrestling game in a beach

Wrestling is the most popular sport in Senegal while in the world, football is still ahead. This popularity of wrestling is explained by the over-inflated media coverage but also by the huge amount of money that a wrestler can win in seconds in fight. Children practice wrestling to mimic wrestlers, to be like them one day and be able to support their families. They are exposed to accidents because of their fragility and their lack of technical mastery of the game. In our series, wrestling game injuries mainly affect older children. According to Thélot [4], it is at that age that physical activity is the most intense, with a desire for autonomy, resulting in aggressiveness responsible for game-related injuries. Injuries due to wrestling are almost exclusively encountered among boys. Three aspects may explain this male predominance: the physical characteristics of boys, the almost non-existence of female wrestling in Dakar, and the fact that, in our context, girls often help their mothers with household work and are less exposed to risks of game injuries. In Western studies, game injuries occur most frequently at school and in playgrounds [5, 6]. In our study, wrestling game injuries occur mainly at home and in the street. Home is the most immediately accessible playing ground for children; in addition, wrestling doesn’t require facilities or equipment, children practice it in the garden or the yard. When children can not play at home, they transform the street into a playground unlike what happens in Western countries where there are safe playing areas. We find out that children with wrestling game injuries are mainly from Suburban Dakar. The predominance of this geographical area is due to the influence of the coaching facilities’ proximity. Children are regularly in contact with the wrestling champions who are social success role models. They practice wrestling as a game, giving themselves the names of their idols. Dakar suburbs are characterized by extreme poverty of the population that is why the majority of wrestling game injury victims are from low socioeconomic status families. Whatever the nature of the lesion, the predominance of upper limb location is obvious. In traditional Senegalese wrestling, the goal is to throw the opponent on the ground, which can therefore cause frequent falls and landing on this part of the body. The elbow is the most exposed location for lesions, whatever their nature. The supracondylar, medial epicondyle and humeral shaft fractures are the most frequent fractures in wrestling game injuries. Fractures of the distal quarter of the two bones of the forearm, which are the most common in children [7, 8], are exceptional in this case. Wrestling is a great purveyor of elbow fractures and reverses the order of fracture injuries’ frequency in children. For other injuries, three-quarters of contusions are located on the elbow, and all other types of injuries (dislocations, sprains, wounds) practically occur on the elbow. This high frequency localization on the elbow is explained by the fact that this region is often used as shield and therefore serves as cushion during falls. We do not find maxillofacial trauma, as in the study of Seck [9] conducted in adults practicing wrestling as a sport. In fact, children who practice wrestling favor blow-free fights and do not make use of punches as adults do. We also note that all lesions are closed injuries. We can explain this result by the tendency of children to choose sandy wrestling grounds to practice this game (Figure 5).

## Conclusion

Wrestling game injuries in Dakar occur among older children from the suburbs, and living next to the great wrestling champions. Wrestling game cause injuries, consisting mostly of elbow fractures.

### What is known about this topic

- In the literature, freestyle and Olympic wrestling are mostly practiced;
- Football is the largest providers of home and leisure injuries in children;
- Lesions are dominated by contusions.

### What this study adds

- The type of wrestling imitated by children exists only in Senegal;
- Wrestling game is the most popular sport in children at Dakar;
- Lesions are dominated by fractures in our context.
